# SuperLearner approach for predicting imminent risk of fracture in older Chinese patients with newly diagnosed osteoporosis based on their routine blood test markers

**DOI:** 10.1186/s12891-026-09768-z

**Published:** 2026-04-21

**Authors:** Bailu Chen, Jinshan Zhang, Yangzhen Fang, Chao Liu, Ming Chen

**Affiliations:** 1 Information Department, Jinjiang Municipal Hospital (Shanghai Sixth People’s Hospital Fujian), Jinjiang City, Quanzhou, Fujian 362200 China; 2 Department of Orthopedic Surgery, Jinjiang Municipal Hospital (Shanghai Sixth People’s Hospital Fujian), Jinjiang City, Quanzhou, Fujian 362200 China; 3 Clinical Research Center for Orthopaedic Trauma and Reconstruction of Fujian Province, Jinjiang City, Quanzhou, Fujian 362200 China; 4 Yidu Cloud Technology Inc, Beijing, 100083 China; 5 Nanjing YiGenCloud Institute, Nanjing, 211899 China

**Keywords:** Osteoporosis, Older adults, Imminent fracture, Machine learning, SHapley Additive exPlanation

## Abstract

**Purpose:**

In this study, we aimed to develop 2-year fracture prediction models using machine learning approaches based on routine blood test markers in patients newly diagnosed with osteoporosis.

**Methods:**

We retrospectively analyzed data from 497 elderly patients (121 men and 376 women) newly diagnosed with osteoporosis without prior fractures at Jinjiang Municipal Hospital between 2011 and 2023. The dataset was randomly divided into training and validation cohorts. Various readily available medical characteristics at the initial diagnosis of osteoporosis were used to develop the prediction models employing the linear, nonlinear, and SuperLearner approaches. Model performance was assessed using the area under the curve (AUC) and the SHapley Additive exPlanation was used to interpret the best-performing models.

**Results:**

In total, 229 (46.1%) of the 497 participants experienced fractures (at any site) within 2 years of osteoporosis diagnosis. The SuperLearner model demonstrated the highest AUC (0.677, 95% confidence interval: [0.591, 0.762]) for predicting the outcome of interest in the internal validation set. Age at diagnosis, serum sodium levels, and other factors emerged as the top-ranked features. Older patients with hyponatremia, low aspartate transferase levels, and hypocalcemia have an increased imminent risk of fracture.

**Conclusions:**

We successfully developed and validated a fracture risk prediction model for patients newly diagnosed with osteoporosis. The combination of routine blood test markers and advanced machine learning algorithms has demonstrated a predictive value for forecasting fractures in individuals with osteoporosis.

**Supplementary Information:**

The online version contains supplementary material available at 10.1186/s12891-026-09768-z.

## Mini abstract

This study developed a model to predict fractures in patients with newly diagnosed osteoporosis. Leveraging routine blood test data and machine learning, the SuperLearner model demonstrated high accuracy in identifying key predictors. This innovation offers promise for improving fracture prevention and management and enhancing overall patient care.

**Supplementary Information:**

The online version contains supplementary material available at 10.1186/s12891-026-09768-z.

## Introduction

Osteoporosis is a disease characterized by increased bone turnover and decreased bone mass with associated skeletal fragility and is common in people aged over 50 years [1, 2]. Fracture, one of the most severe complications of osteoporosis, is a well-recognized health problem, as it is often unrecognized until the late stages [2]. However, once diagnosed, it confers significant medical costs and quality-of-life decrements (e.g., reduced mobility) [3]. This situation is worsened because older adults are at a higher risk of developing fractures, and an ever-increasing aging population is expected globally [4, 5]. Therefore, a precise diagnosis and early prediction is vital.

There is an emerging literature on fracture prediction. One of the most well-known tools is FRAX, a widely used tool developed by the World Health Organization for assessing the risk of fractures in individuals [6]. It estimates the 10-year probability of a major osteoporotic fracture (including hip, spine, forearm, or shoulder) and specifically a hip fracture [7]. Precisely, the FRAX tool considers several factors that contribute to fracture risk, including age, sex, body mass index (BMI), previous fractures, parental history of hip fracture, current smoking status, glucocorticoid use, alcohol intake, presence of certain medical conditions such as rheumatoid arthritis. This tool also incorporates bone mineral density (BMD) measurements if available [8]. In addition to general risk prediction models, such as the FRAX, models have been developed for specific populations (based on specific factors). Liu et al. developed a fracture risk prediction model specifically for older Chinese patients with osteoporosis using the hybrid model, the final hybrid extreme gradient boosting (XGBoost) and the support vector machine model, which incorporated 20 critical features (blood and urine tests) and achieved a predictive accuracy of 0.9 [5]. Khalid et al. developed and validated prognostic models for predicting imminent fractures in patients with a recent fracture or starting oral bisphosphonate therapy, and with selected predictors focusing on therapy, the final model’s discriminatory ability for any fracture was low to acceptable (0.53–0.60) [9]. Barron et al. previously explored the determinants of imminent fracture risk in postmenopausal women with osteoporosis and found that prior fracture, lower physical functioning, and recent falls all directly or indirectly influence imminent fracture risk [10]. Using US claims data, Bonafede et al. identified factors associated with imminent risk for fracture with emphasis on specific comorbidities, such as psychosis, Alzheimer’s disease, and central nervous system diseases [11].

The FRAX focuses exclusively on long-term fracture risk (10-year time horizon). However, the literature highlights that considering the imminent risk (within the next 1–2 years) of fracture has significant implications for making informed treatment decisions [3, 12]; therefore, the imminent risk is now attracting more attention compared with long-term fracture, which would happen in approximately half of the older adult populations and has a mixed etiology.

Fractures are often unrecognized until the late stages; therefore, it may be difficult for patients newly diagnosed with osteoporosis to realize the risk of fracture [2]. However, if a fracture occurs, we can only administer symptomatic treatment in most scenarios and wait for the bone to recover slowly [13]. Therefore, incorporating easily accessible clinical variables into the model and developing it as a screening tool to identify newly diagnosed patients with osteoporosis who are at a high risk of fracture is essential. Moreover, patients initially diagnosed with osteoporosis during hospital visits typically undergo routine clinical tests that provide readily available measurements, making risk estimation more feasible than routine or regular on-site screening. Thus, given the elevated fracture risk in patients with osteoporosis, this approach (imminent fracture risk prediction in patients newly diagnosed with osteoporosis) emphasizes the necessity for fracture risk prediction at a point or time where necessity meets feasibility.

Bone turnover markers, including BMD, are widely acknowledged as risk factors of fracture [14–17]; however, studies have demonstrated that relying solely on BMD testing is not sufficiently reliable for predicting fracture development [8]. Moreover, limitations such as high cost, ionizing radiation exposure, and low mobility associated with BMD testing restrict its clinical applicability [18]. Notably, some studies have attempted to improve the prediction model performance using recent fractures as predictors [12, 19]. However, recent fractures are well-known risk factors, reducing the urgency of risk prediction in patients with recent fractures, as all patients with recent fractures inherently require fracture prevention measures. Therefore, relying primarily on routine blood test markers to build a prediction model for fractures and discussing the underlying mechanism revealed by the association is essential.

Previous studies in this field have primarily focused on risk factor analyses using methods such as case–control studies; only a few studies have employed machine learning frameworks that include validation procedures [20], and even fewer have investigated the association between blood markers and fracture risk [21]. This is likely because individual routine blood test markers are suspected to have limited predictive value for future fractures. However, when combined, these markers may complement one another and facilitate prediction. Therefore, advanced models, such as ensemble machine learning algorithms, can help uncover the complex interactions between these variables.

**Fig. 1:**
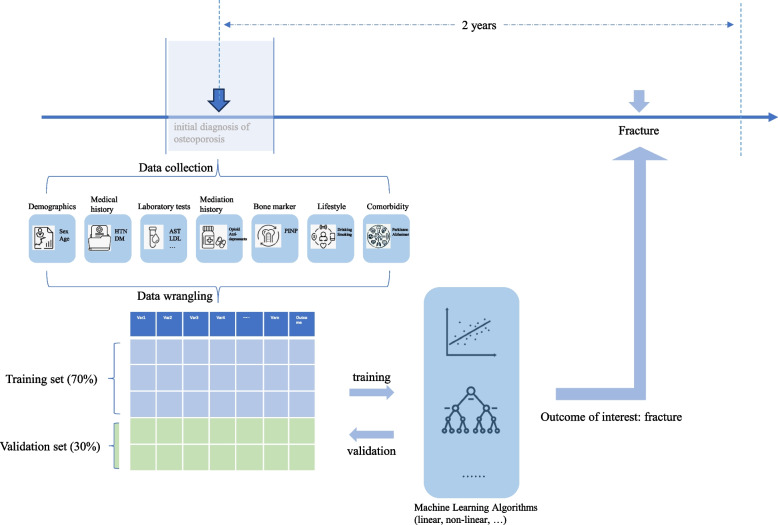
Overview of the machine learning approach for predicting imminent risk of fracture in newly diagnosed osteoporosis patients

To address the gaps in existing research, we aimed to overcome certain challenges by developing a prediction model for the imminent risk of fracture in newly diagnosed osteoporosis patients without recent fractures, utilizing their routine blood test markers **(**Fig. 1**)**. By employing a machine learning approach, we achieved modest-to-good discriminative ability for predicting fractures. Furthermore, we utilized SHapley Additive exPlanations (SHAP) to enhance the interpretability of the model, allowing us to understand each feature’s contribution to the prediction outcome at the individual patient level. This improves our understanding of the factors influencing fracture risk and provides avenues for future research. In addition, the identification of novel markers associated with the imminent risk of fractures opens up new possibilities for further investigation in this field.

## Methods

### Study design and population

This retrospective cohort study included patients initially diagnosed with osteoporosis at Jinjiang Municipal Hospital between 2011 and 2023. All patients with at least one occurrence of osteoporosis were screened using the International Classification of Diseases (ICD)−9 or ICD-10 codes in the outpatient and inpatient departments. The baseline was established as the visit corresponding to the initial documented diagnosis of osteoporosis in each patient.

The inclusion criteria were: (1) (baseline) age ≥ 50 years; (2) at least one hospital visit (regardless of visit type) within 2 years of the baseline visit.

The exclusion criteria were as follows: (1) recently documented fracture (in the previous 24 months); (2) conditions including osteogenesis imperfecta, bone metastasis, Paget’s disease, endocrine dysfunction, chronic kidney disease, and malignancy, which significantly impact bone metabolism; and (3) receiving glucocorticoids, vitamin D, bisphosphonates, calcitonin, or other drugs that affect bone metabolism.

### Candidate predictor variables and outcome

Based on clinical expertise, evidence from previous studies [21, 22], and data availability (routinely measured characteristics), we identified a set of potential predictor candidates that were either clinically relevant or data-driven during study design. These variables were extracted from electronic medical records at patients’ baseline visits, that is, the visit corresponding to the initial documented diagnosis of osteoporosis in each patient. Original candidate predictor variables were classified into the following categories: (1) demographics (sex and age at baseline); (2) medical history (e.g., hypertension); (3) (routine blood) laboratory tests (e.g., aspartate transferase [AST]); (4) medication history (e.g., opioid and antidepressants); (5) bone (turnover) markers (e.g., procollagen type I N-propeptide); (6) lifestyle (e.g., drinking); (7) comorbidity (e.g., dementia), and others (e.g., BMI). All baseline predictor variables were collected during the patient’s baseline visit, and the value closest to the time of diagnosis was selected if there were multiple testing values for one parameter.

The primary outcome of interest was a documented fracture in the inpatient or outpatient records in the above databases at any site (except the face, skull, and digits) during the follow-up period, defined as two years within the initial diagnosis of osteoporosis.

### Modeling

To prepare the data for modeling, patients with outliers in any predictor variable, as determined by the doctors with clinical expertise, were excluded. Variables with a missing data rate exceeding 70% in the initial cohort were excluded prior to analysis. This threshold was applied to minimize potential bias and ensure that the retained variables contained sufficient information for reliable statistical estimation and model training. Subsequently, patients with missing data for over 50% of the remaining variables were excluded from the study. To handle missing values, a multivariate imputation strategy was used to prepare the data matrix for modeling [23]. The variables utilized in the analyses were converted to numeric or binary values; for instance, 1 represented men and 0 represented women. The primary outcome variable, or the prediction target, was converted to a binary format: zero indicating a negative result (no fracture) and one indicating a positive result (fracture present).

The cohort dataset was divided into a training set (70%, N = 347) and a validation set (30%, N = 150) using a random assignment process before model development. Notably, various machine learning algorithms, including linear models, such as multivariate logistic regression and least absolute shrinkage and selection operator, nonlinear models, such as random forest and XGBoost, and SuperLearner, have been employed to construct prediction models [24]. The differences between these algorithms are presented in Table S1 and the rationale for prioritizing the algorithms is further discussed in the Discussion section. The optimal parameters of the machine learning algorithms were determined by cross-validation with the training set. A performance evaluation was conducted using the validation set, and the model exhibiting the largest area under the receiver operating characteristic curve (AUC) was selected as the final best-performing model. To further evaluate the uncertainty of the model and reinforce the robustness of SuperLearner, resampling techniques, including random partitioning and bootstrapping, were applied. Model performance was assessed on both randomly partitioned validation sets and bootstrapped ‘out-of-bag’ samples [25]. These steps were incorporated to assess the generalization capability of the model across different subsets of data. To interpret the results of the selected model, SHAP was used to quantify the contribution of each variable to the prediction [26].

### Model evaluation and validation

The performance of the best-performing model (SuperLearner) was evaluated using the AUC. The accuracy of the optimal cutoff value was assessed using sensitivity, specificity, and positive and negative predictive values (PPV and NPV, respectively).

### Statistical analysis

Unpaired two-tailed t-tests and Wilcoxon tests were used to compare the distributions of continuous variables. For variables that did not exhibit a normal distribution, comparisons were made based on median and quantile values. The chi-square test was used to assess the relationships between categorical variables, such as the balance of labels between the training and validation sets. Missing values were imputed through multivariate imputation using the chained equations package. The SuperLearner model was implemented using the ‘SuperLearner’ R package. Statistical significance was set at a p-value of 0.05, and all statistical analyses were performed using R version 4.0.1.

## Results

### Patient characteristics

Overall, 497 patients were included in this study, and the initial diagnosis of osteoporosis was evenly distributed over 12 years (2011–2023). The median age of the patients was 73 years (interquartile range [IQR], 65–79 years), and 75.7% of them were women. Within 2 years of the initial diagnosis of osteoporosis, 229 (46.1%) patients had experienced fractures.

### Predictor variables and outcomes

After filtering for missing rate-based data, we identified 497 patients and 24 variables (23 predictor variables and one outcome variable). A list of variables and a comparison of the patients with and without fracture are shown in Table 1 (Notes: variables exhibiting zero or approximately zero variance across the selected patients, such as comorbidities, were omitted from modeling but retained for descriptive analysis). The lymphocytes, calcium, alanine transaminase, and serum sodium levels were significantly higher in patients without fractures than in those with fractures. Patients with fractures had significantly higher age, neutrophil count, and proportion of men. No significant differences were found in the other variables between the groups. All clinical variables were well-balanced between the training and validation sets (Table S2).

**Table 1:** Demographic, clinical, and biological characteristics of the enrolled patients with and without fracture

Characteristics	Fracture (*N* = 229)	Non-Fracture (*N* = 268)	*p*-value
Demographics			
Gender			0.014
Female	161	215	
Male	68	53	
Age (years)	74.50 (9.31)	70.19 (8.88)	< 0.001^#^
Lifestyle			
Drinking			0.076
Yes	2	10	
No	227	258	
Smoking			0.737
Yes	19	19	
No	249	210	
Laboratory tests			
Lymph (%)	21 (14.8–29.3)	25.15 (19.1–31.9)	< 0.001
Neut (%)	67.7 (59.5–74.9)	64.2 (56.7–71.63)	0.002
Ca (mmol/L)	2.27 (2.16–2.36)	2.31 (2.22–2.4)	< 0.001
ALT (U/L)	15 (11–21.8)	17.1 (13–23.2)	0.006
Na (mmol/L)	138.9 (135–141)	139.4 (137.56–141)	0.012
ApoB (g/L)	0.9 (0.69–1.135)	0.858 (0.69–1.06)	0.167
Crea (umol/L)	65.79 (52.61–81)	61.1 (52.7–74.15)	0.066
HDL (mmol/L)	1.31 (1.07–1.68)	1.315 (1.06–1.635)	0.910
AST (U/L)	20.5 (16–25.2)	20.9 (17–26.7)	0.150
ALBGLO (ratio)	1.33 (1.14–1.57)	1.41 (1.20–1.57)	0.106
ALP (U/L)	83 (66–105)	79.55 (64–100)	0.204
UA (umol/L)	309 (250.9–380.8)	322.35 (257.325–398.775)	0.219
LDL (mmol/L)	2.72 (2.22–3.46)	2.67 (2.20–3.37)	0.581
TC (mmol/L)	4.7 (3.96–5.51)	4.645 (3.928–5.553)	0.696
TBIL (umol/L)	10.7 (8.19–13.8)	10.75 (8.075–13.65)	0.749
apoAI (g/L)	1.16 (0.94–1.39)	1.16 (0.93–1.38)	0.928
TG (mmol/L)	1.14 (0.81–1.9)	1.185 (0.878–1.66)	0.742
Medical history			
Diabetes	49 (21.4%)	55 (20.5%)	0.898
Hypertension	100 (43.7%)	136 (50.7%)	0.138
Comorbidities*			
Parkinson's disease	0 (0%)	1 (0.37%)	> 0.99
Alzheimer's disease	0 (0%)	1 (0.37%)	> 0.99
NSAIDs-induced gastrointestinal bleeding	0 (0%)	0 (0%)	> 0.99

*Lymph* lymphocyte, *Neut* neutrophil, *Ca* calcium, *ALT* alanine transaminase, *Na* serum sodium, *ApoB* Apolipoprotein B, *Crea* Creatinine, *HDL* High-density lipoprotein cholesterol, *AST* aspartate aminotransferase, *ALBGLO* albumin to globulin ratio, *ALP* alkaline phosphatase, *UA* uric acid, *LDL* low-density lipoproteins, *TC* total cholesterol, *TBIL* total bilirubin, *apoAI* Apolipoprotein A-I, *TG* triglyceride, *NSAIDS* nonsteroidal anti-inflammatory drugs

^#^Age is the only variable that followed a normal distribution; thus, it is presented as the mean (SD)

^*^Due to low variance, these variables were excluded from modeling, but not from descriptive analysis

### Model development, evaluation, and validation

After imputing the missing values, no significant outliers were observed in the feature sets. No significant differences in the incidence of fractures were observed between the training and validation sets (46.1% vs. 46.0%, *p* > 0.99).

**Fig. 2:**
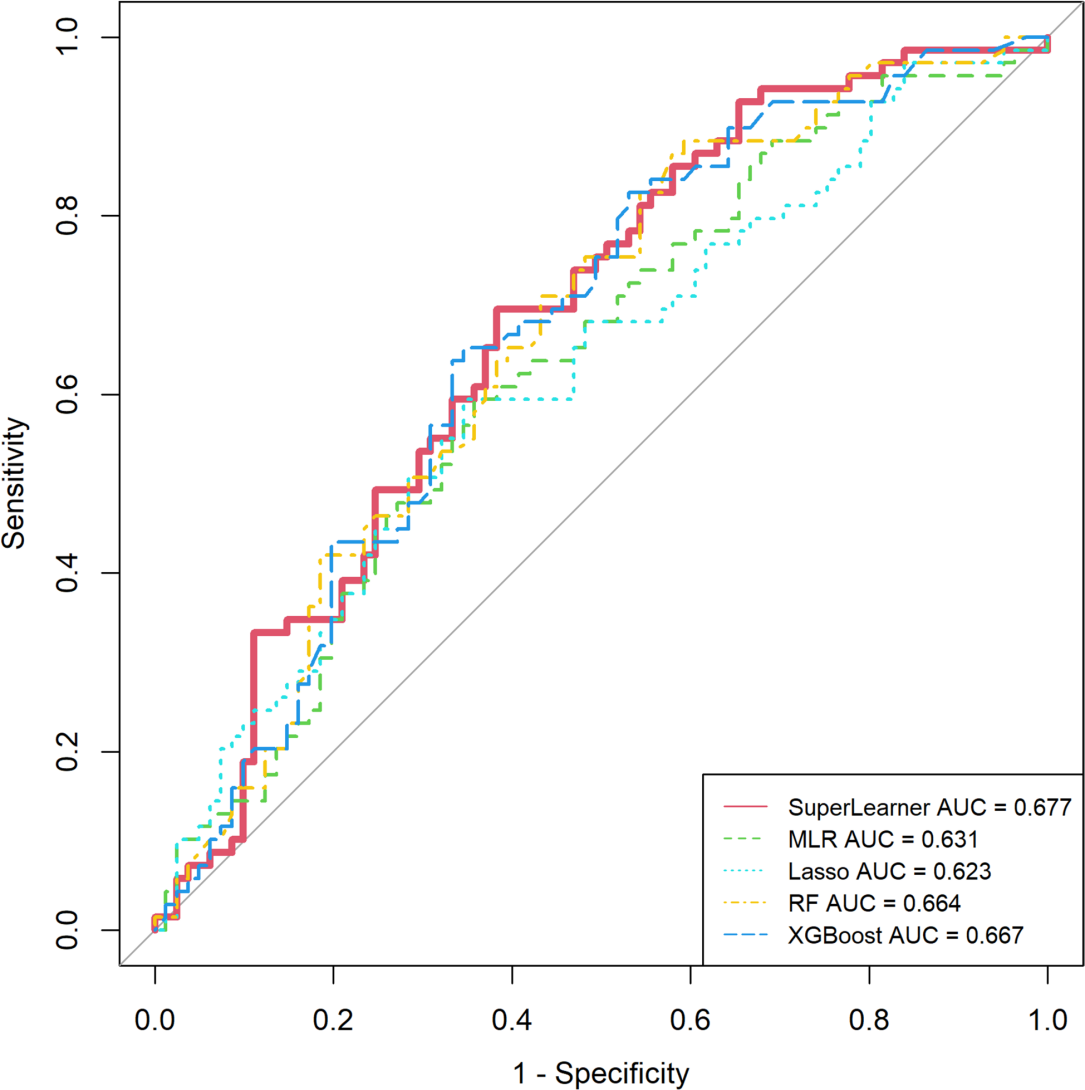
Receiver operating characteristic curves for evaluating the discrimination ability of the model. Notes: SuperLearner had the highest area under the curve compared with the other models

All predictor variables (N = 23) were used as inputs for the five machine learning algorithms to predict the imminent risk of fracture. The discrimination abilities were compared **(**Table S3), and SuperLearner had the highest AUC (0.677, 95% confidence interval [CI] 0.591–0.762), and was thus selected as the final best-performing model (Fig. 2, Table S3). The median AUC for the 100 randomly partitioned validation sets was 0.657 (IQR [0.635, 0.688]) (Table S4), while the median AUC for the 100 bootstrapped ‘out-of-bag’ samples was 0.660 (IQR [0.640, 0.680]) (Table S4). In the validation cohort, the sensitivity, specificity, PPV, and NPV for differentiating the fractures were 0.6087, 0.6420, 0.5915, and 0.6582, respectively (Table 2).

**Table 2:** Prediction accuracy of the SuperLearner estimating the risk of imminent fracture in patients with newly diagnosed osteoporosis in the training and validation cohorts

**Variable**	**Value (95% C.I.)**	
	**Training cohort** **(*N* = 347)**	**Validation Cohort** **(*N* = 150)**
#positive events	160 (46.1%)	69 (46.0%)
AUC	0.9424 (0.9203, 0.9646)	0.6767 (0.591, 0.7624)
Cutoff probability	0.45	0.45
Sensitivity, %	88.12 (82.08, 92.70)	60.87 (48.37, 72.40)
Specificity, %	84.49 (78.49, 89.36)	64.20 (52.77, 74.55)
PPV, %	82.94 (76.43, 88.27)	59.15 (46.84, 70.65)
NPV, %	89.27 (83.75, 93.41)	65.82 (54.29, 76.13)
Positive likelihood ratio	5.6825 (4.0473, 7.9785)	1.7001 (1.2009, 2.4069)
Negative likelihood ratio	0.1405 (0.0917, 0.2153)	0.6095 (0.4355, 0.8531)

*AUC* Area under the curve, *CI* confidence interval, *PPV* Positive predictive value, *NPV* Negative predictive value

### Explanation of risk factors

**Fig. 3:**
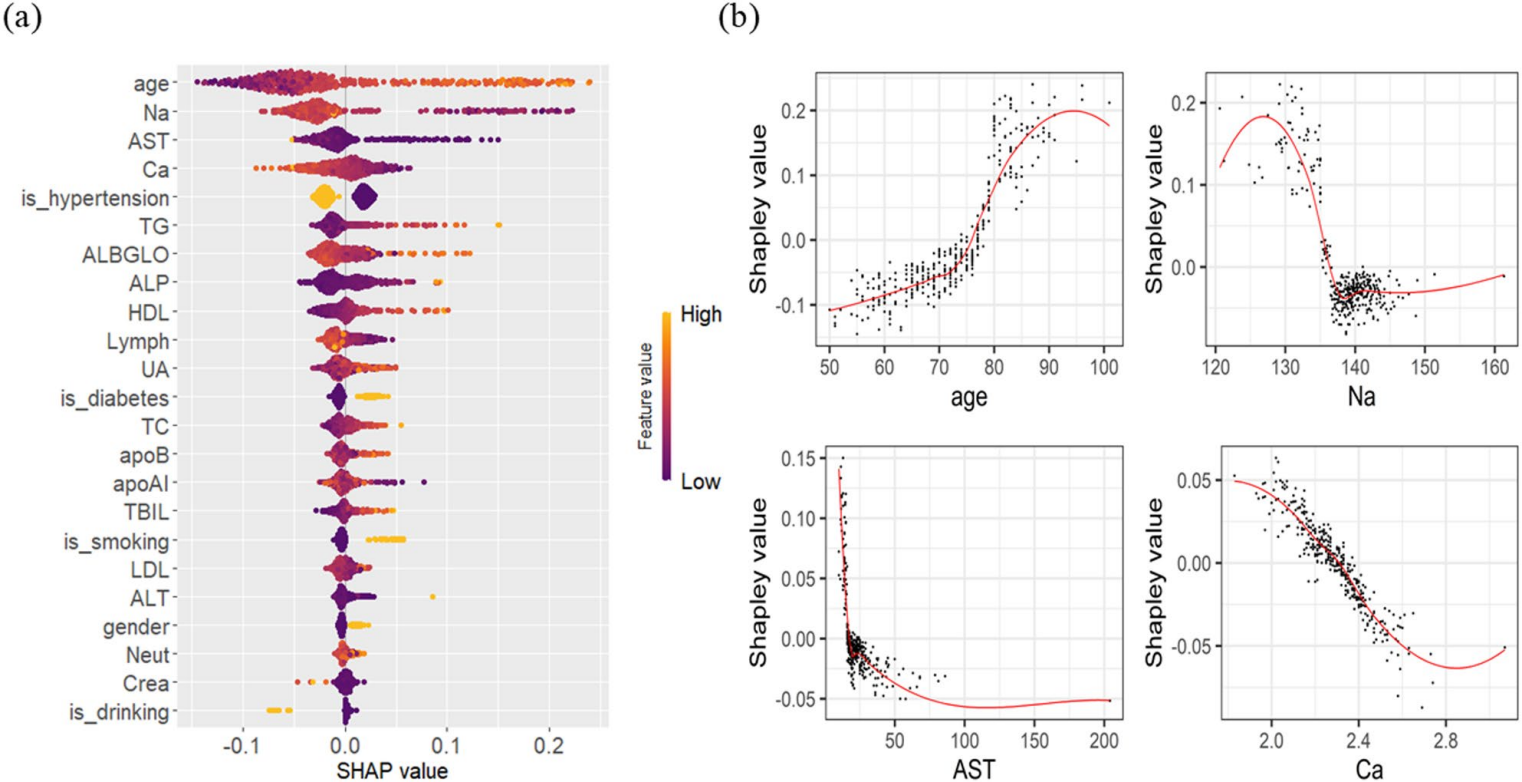
Model interpretability assessed using SHapley Additive exPlanation. **a** The SuperLearner bee swarm plot depicts each variable’s importance for predicting the risk of imminent fracture in newly diagnosed osteoporosis. Footnote: One dot per patient per feature is colored according to the attribute value, where orange and purple represent the higher and lower values, respectively. **b** SHapley Additive exPlanation dependence plot of SuperLearner (selected top four features), depicting how a single variable affects prediction

SHAP was used to interpret the results of SuperLearner by computing the contribution of each variable to the prediction [27]. A SHAP summary plot (beeswarm) is shown in Fig. 3. The importance plot ranked the variables contributing to fracture risk prediction, with patients' baseline age at the forefront, followed by serum sodium, AST, and calcium levels, with additional variables also included in the ranking.

The SHAP dependence plots of the four selected variables included in SuperLearner are shown in Fig. 3 (the remaining 19 plots are shown in Fig. S1). Higher SHAP values are associated with an increased risk of fracture. Older patients with lower serum sodium (hyponatremia), AST, and calcium (hypocalcemia) levels have an increased imminent risk of fracture. The feature value associated with zero SHAP can be used as a reference to determine the desired value of the variable, acting as a tipping point to distinguish between positive and negative contributions to the risk of imminent fracture.

## Discussion

The present study used the data of hospital-visiting patients initially diagnosed with osteoporosis without a recent fracture and used SuperLearner and SHAP to uncover the complex relationships between predictors and fracture occurrence. Compared with existing studies on developing prognostic models to predict fractures in patients with osteoporosis [2, 5, 11, 21], we focused on predicting the imminent risk upon the initial diagnosis of osteoporosis using advanced machine learning methods based on routine blood test markers. The study design, ensuring real-world relevance, improved clinical applicability, and revealed associations between new markers and the imminent risk of fracture, offer new research avenues.

The association between osteoporosis and increased fracture risk is well-established [20]; however, limited attention has been paid to setting a prediction window to estimate the risk as soon as possible, such as upon the initial diagnosis of osteoporosis. Additionally, it is crucial to differentiate between imminent and long-term risk [28], as an elevated imminent risk may require a different treatment approach from the fracture risk that accumulates or extends over a longer period [3]. A prior fracture inherently increases the risk of subsequent fractures [21]; therefore, the focus on predicting the imminent fracture risk in individuals without recent fractures is of particular interest [11]. This study design enhances the practicality and validity of the final predictive model by focusing on predicting the imminent risk at the time of initial osteoporosis diagnosis.

Notably, several well-known risk factors for fractures exist, including previous falls/recent fractures, medication use (glucocorticoids), and bone turnover markers (e.g., BMD) [20]. Among these factors, objectively measurable variables, such as BMD, are the most commonly used [21]. However, traditional approaches based solely on BMD measurement are unsuitable for population screening due to cost and availability issues [8, 18]. This raises the important question of whether routine blood test markers can provide better predictive value. Previous studies have not fully explored the predictive potential of blood test markers because it is suspected that a single marker or a simple linear combination of multiple markers may not adequately capture the predictive value or achieve satisfactory sensitivity. From a modeling perspective, machine learning algorithms can be classified into linear and nonlinear categories, with the latter including bagging, boosting, and stacking, which are well-represented by the algorithms used in this study. However, single linear or nonlinear algorithms may not be sufficient to capture the complex, interacting relationships among the many variables that influence fracture risk. Therefore, recognizing the complexity of multiple interacting characteristics that potentially influence fracture risk, this study conducted analyses using linear, nonlinear (including ensemble learning algorithms), and SuperLearner models. Comparisons of model performance demonstrated the superior performance of the SuperLearner model over the nonlinear models, which generally outperformed the linear models in the validation set (Fig. 2, Table S3).

Although direct comparisons may be limited due to differences in study population, outcome definition, and prediction window, our model demonstrated comparable performance (AUC close to 0.7) to previous fracture prediction models, including those using less readily available predictors such as bone mineral density (BMD) [21]. By integrating routinely available clinical and laboratory variables and applying an ensemble machine learning approach, our model achieved comparable discrimination for predicting imminent fracture risk at an earlier stage of disease assessment. Furthermore, the incorporation of SHAP analysis enhanced interpretability, enabling identification of key predictors and individualized risk contributions.

When constructing a more complex model, greater interpretability is crucial. One strength of SHAP is its ability to provide individualized explanations, offering a higher level of granularity in understanding the model’s predictions [29]. Combining the SuperLearner model with SHAP confirmed the prognostic value of several common risk factors. In this study, the analysis revealed that age, serum sodium, AST, and calcium levels were correlated with fracture risk (Fig. 3b). Previous studies have also identified these markers as critical variables for predicting fracture risk [30–32]. Specifically, older patients with hyponatremia and low AST and serum calcium levels were found to have a higher imminent risk of fracture. Furthermore, as the SHAP dependence plot is an accumulation of individualized explanations, it depicts the general association between variables and the risk of fracture and reveals a ‘desired value’ (safe range) for the variables. For example, the tipping point for age was in the range of 75–80 (Fig. 3b), which is consistent with the results of previous studies.

In the present study, low baseline serum sodium levels (< 135 mmol/L) were associated with a high imminent risk of fracture, consistent with previous reports linking hyponatremia to an increased risk of hip fractures in older adults [31]. Hyponatremia may contribute to decreased BMD, compromised bone strength, and a higher likelihood of falls due to muscle weakness and impaired coordination [33, 34]. It has also been associated with altered calcium and phosphate levels, which are crucial for bone metabolism, although the roles of parathyroid hormone and vitamin D in this process remain unclear [35, 36]. Severe hyponatremia (< 120 mmol/L) can further impair balance and cognition, increasing fall risk [37]. These findings underscore the importance of recognizing and managing hyponatremia in older adults to reduce fracture risk through regular sodium monitoring and fall prevention strategies.

Our study found that lower AST levels (within the normal range of 0–40 U/L) were associated with a higher imminent fracture risk. Although previous studies have reported inconsistent findings, this association may reflect overall poor health rather than a direct causal effect. Low AST levels can indicate malnutrition or chronic conditions affecting liver function and nutrient metabolism, such as vitamin B6 deficiency, which may impair bone and muscle health and increase frailty [38, 39]. Identifying and managing the underlying causes of low AST levels through comprehensive assessment of nutritional and health status may help reduce fracture risk in older adults.

We observed a positive association between dyslipidemia, inflammation, and imminent fracture risk, reflected by positive SHAP values for elevated triglycerides, total cholesterol, Apolipoprotein B (ApoB), diabetes status, and reduced Apolipoprotein A-I (ApoA-I) (Fig. S1). Consistent with previous studies [30, 40–42], dyslipidemia may adversely affect bone metabolism and microarchitecture, increasing fracture susceptibility, particularly in older adults. It also contributes to atherosclerosis, which can impair bone perfusion and nutrient delivery, and to vascular calcification, which reduces arterial elasticity and further compromises bone health [43]. Moreover, dyslipidemia is frequently accompanied by chronic low-grade inflammation and oxidative stress—evidenced by altered albumin/globulin ratios, lymphocyte depletion, and neutrophil elevation—that can promote bone loss and impair repair processes [44]. Given the potential confounding effects of comorbidities and individual differences, further research is needed to clarify the mechanisms linking dyslipidemia to fracture risk in the elderly.

The findings of the present study have several important clinical implications. First, utilizing a real-world study design alongside strict inclusion and exclusion criteria, our model demonstrated a moderately good AUC, signifying a strong discriminatory capability based on readily available characteristics. Second, the SHAP-generated dependence plots were used to establish index-specific fracture risk thresholds. These findings, when corroborated with mechanism validation, can effectively guide clinical decision-making and enable the implementation of targeted interventions.

This study has some limitations. First, the analysis relied on patient data from a single center, potentially introducing selection bias and limiting the heterogeneity of certain variables. For instance, medication and medical histories were excluded from the analysis because of the lack of heterogeneity (Table 1). These variables can influence fracture outcomes; however, their limited variability restricts their usefulness in developing an early fracture risk model. Second, although notable associations were identified in this study, it is important to note that these correlations do not imply causation. Further investigations are necessary to elucidate the mechanisms underlying these relationships.

## Conclusions

Overall, the present study employed real-world data and an innovative analytical approach to construct a SHAP-based explainable SuperLearner model. This model proficiently forecasts the imminent fracture risk among patients recently diagnosed with osteoporosis during hospital visits. The inclusion of routine blood test markers in the model holds promise for improving patient outcomes by enabling early detection and guiding precise interventions. In future work, we aim to validate the model in multicenter cohorts and through external datasets to assess its generalizability across diverse populations. Further, prospective studies are planned to evaluate the model’s clinical utility and effectiveness in real-world settings, including its impact on clinical decision-making and patient prognosis.

## Supplementary Information

Supplementary Material 1. Fig. S1. SHapley Additive exPlanation dependence plot of SuperLearner (the remaining 19 from the 23), depicting how a single variable affects the prediction.

Supplementary Material 2.

## Data Availability

The datasets used and/or analyzed during the current study are available from the corresponding author upon reasonable request.
