# Supplementary figures and images for: SuperLearner approach for predicting imminent risk of fracture in older Chinese patients with newly diagnosed osteoporosis based on their routine blood test markers
Source: BMC Musculoskelet Disord. 2026 Apr 21;27:527. doi: 10.1186/s12891-026-09768-z (PMC13277268; doi:10.1186/s12891-026-09768-z)

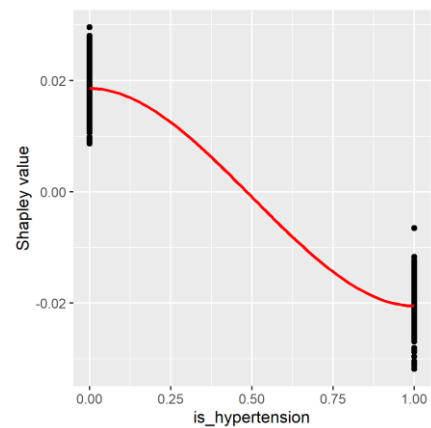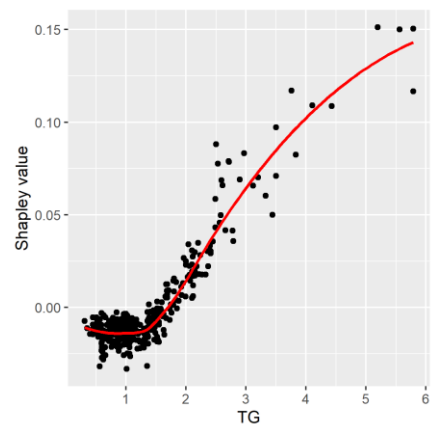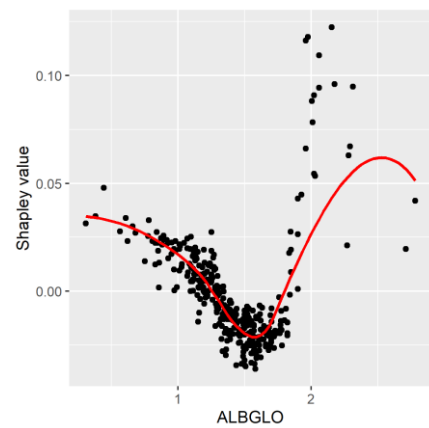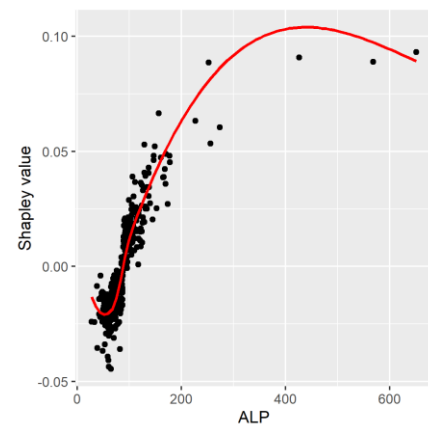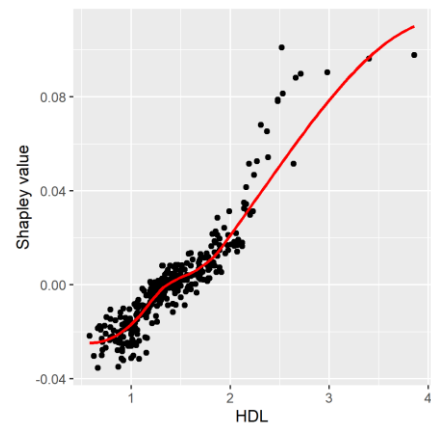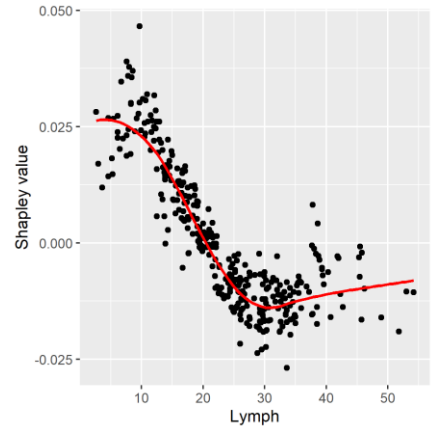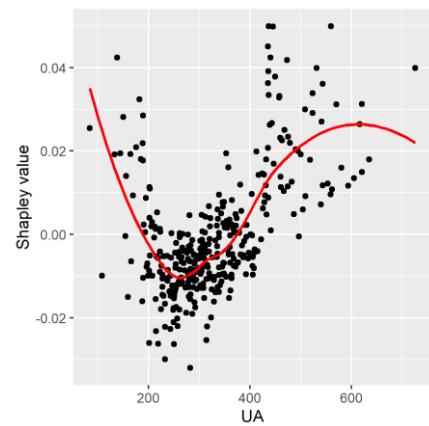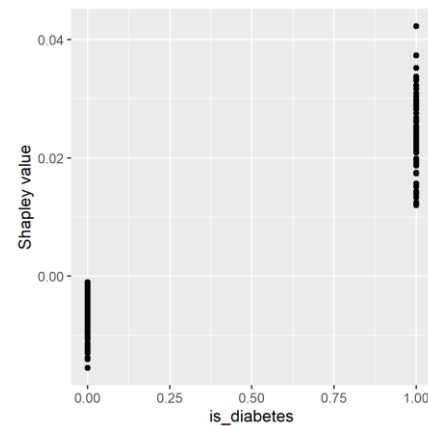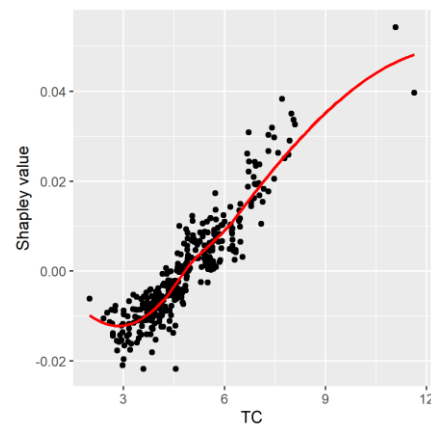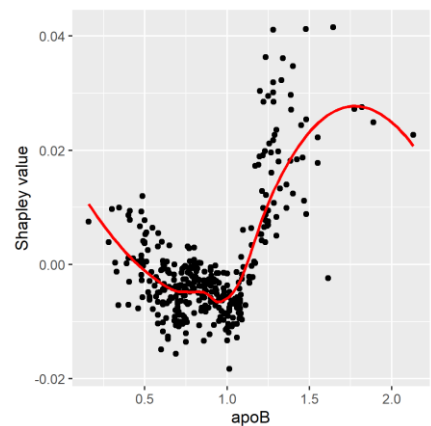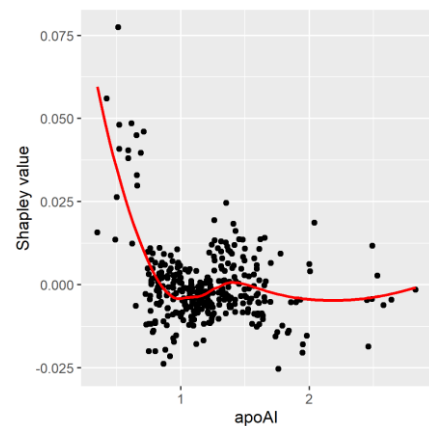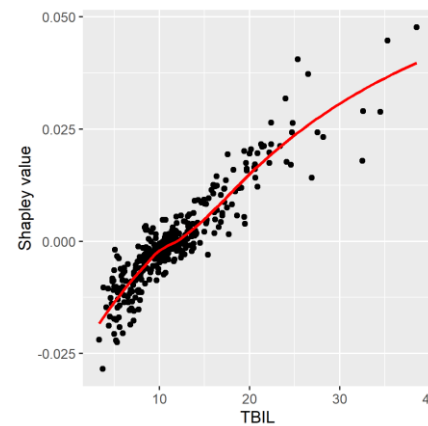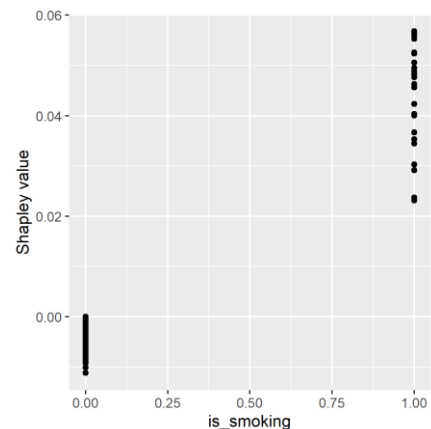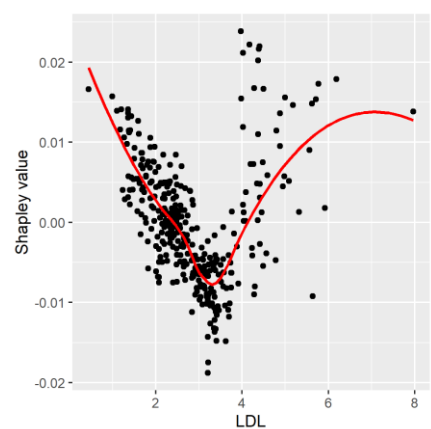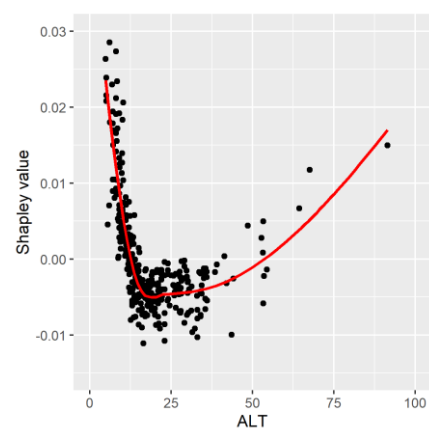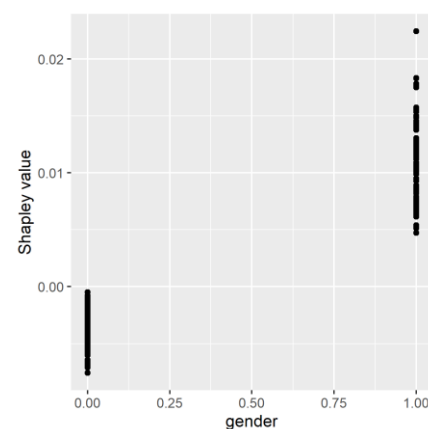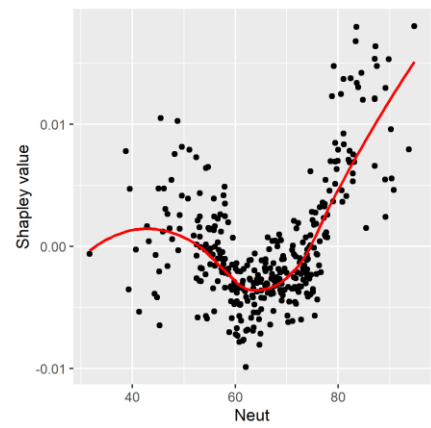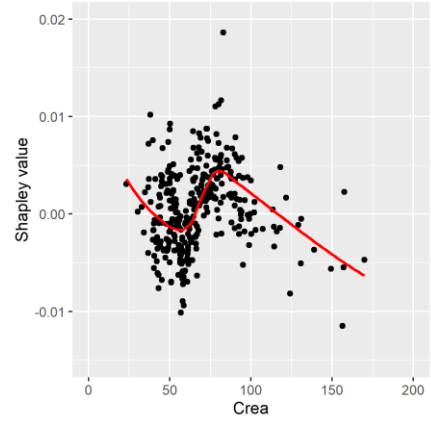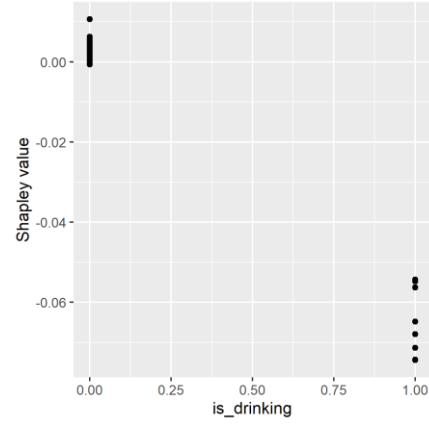

Supplement: Supplementary file 1 — Supplementary Material 1. Fig. S1. SHapley Additive exPlanation dependence plot of SuperLearner (the remaining 19 from the 23), depicting how a single variable affects the prediction. [file 12891_2026_9768_MOESM1_ESM.pdf]
