# Supplementary material for: SuperLearner approach for predicting imminent risk of fracture in older Chinese patients with newly diagnosed osteoporosis based on their routine blood test markers
Source: BMC Musculoskelet Disord. 2026 Apr 21;27:527. doi: 10.1186/s12891-026-09768-z (PMC13277268; doi:10.1186/s12891-026-09768-z)
Supplement: Supplementary file 2 — Supplementary Material 2. [file 12891_2026_9768_MOESM2_ESM.docx]

**Table S1.** Introduction, explanation, and comparison of AI algorithms used in this study.

| **Algorithm** | **Type** | **Introduction** | **Key Strengths** | **Key Weaknesses** | **Common Use Cases** | **Key Parameters** |
| --- | --- | --- | --- | --- | --- | --- |
| MLR (Multivariate Logistic Regression) | Linear model (Logistic Regression) | A logistic regression model used for binary classification with multiple predictor variables. It estimates the probability of a binary outcome based on a linear combination of features. | Simple, interpretable, fast, well-suited for small datasets with linear decision boundaries. | Assumes linearity, may underperform with complex, non-linear relationships or interactions between features. | Medical diagnosis, fraud detection, marketing response prediction. | C (regularization strength) (not applicable in this study) |
| Lasso (Least Absolute Shrinkage and Selection Operator) | Linear model (Logistic Regression) | A regularized version of logistic regression that adds an L1 penalty to encourage sparsity, effectively performing feature selection. | Performs automatic feature selection, prevents overfitting by reducing complexity, works well on high-dimensional data. | Can underperform when many features are correlated, biased if predictors are not well-regularized. | High-dimensional datasets (e.g., genomics, text classification, marketing). | Alpha (regularization strength) |
| Random Forest | Non-linear model  (Bagging) | An ensemble of decision trees trained on random subsets of data. Each tree makes a classification, and the final prediction is the majority vote from all trees. | Handles non-linear relationships, robust to overfitting, works well with noisy data, handles high-dimensional data. | Computationally expensive, harder to interpret, slow to train and predict for large datasets. | Customer segmentation, medical diagnosis, churn prediction. | n_estimators (number of trees)  max_features: (number of features to consider at each split) |
| XGBoost (Extreme Gradient Boosting) | Non-linear model  (Boosting) | A gradient boosting algorithm that builds trees sequentially, each correcting the errors of the previous one, and incorporates regularization to avoid overfitting. | High predictive accuracy, can handle non-linear relationships, fast for large datasets, flexible with custom loss functions. | Can overfit if tuned poorly, requires careful hyperparameter tuning, computationally expensive. | Fraud detection, disease risk prediction, high-performance prediction tasks (e.g., Kaggle competitions). | n_estimators (number of boosting rounds)  max_depth (maximum depth of a tree)  eta (learning rate) |
| SuperLearner | Non-linear model  (Stacking) | Combines multiple models, often including linear and non-linear algorithms, and optimizes the combination of their predictions using a meta-model. | Can improve prediction accuracy by combining the strengths of diverse models, flexible in model choice. | Complex and computationally intensive, requires cross-validation to avoid overfitting, hard to interpret. | Complex prediction tasks, where model diversity leads to better accuracy (e.g., healthcare risk prediction, financial modeling). | V (number of folds for cross-validation)  SL.library (list of base learner functions to use) |

**Table S2**. Demographic, clinical, and biological characteristics of patients in the training and validation cohorts.

| **Characteristics** | **Training**  **(N = 347)** | **Validation**  **(N = 150)** | **p-value** |
| --- | --- | --- | --- |
| **Demographics** |  |  |  |
| Gender |  |  | 0.646 |
| Female | 260 | 116 |  |
| Male | 87 | 34 |  |
| Age (years) | 72.29 (9.43) | 71.92 (9.11) | 0.687^#^ |
| **Lifestyle** |  |  |  |
| Drinking |  |  | > 0.99 |
| Yes | 8 | 4 |  |
| No | 339 | 146 |  |
| Smoking |  |  | > 0.99 |
| Yes | 27 | 11 |  |
| No | 320 | 139 |  |
| **Laboratory tests** |  |  |  |
| Lymph (%) | 23.1 (16.5-30.2) | 23.6 (17.73-32.28) | 0.287 |
| Neut (%) | 65.9 (59.5-74.0) | 65.0 (57.1-71.7) | 0.391 |
| Ca (mmol/L) | 2.29 (2.19-2.38) | 2.30 (2.22-2.37) | 0.350 |
| ALT (U/L) | 16.4 (12-22.95) | 17 (12-22.3) | 0.776 |
| Na (mmol/L) | 139.1 (136.88-141.1) | 139 (137.0-140.7) | 0.796 |
| ApoB (g/L) | 0.86 (0.684-1.075) | 0.9 (0.693-1.056) | 0.666 |
| Crea (umol/L) | 63.2 (52-78.78) | 62.39 (54.93-73.88) | 0.639 |
| HDL (mmol/L) | 1.33 (1.08-1.67) | 1.28 (1.01-1.61) | 0.119 |
| AST (U/L) | 20.4 (17-26) | 20.95 (17-26.38) | 0.723 |
| ALBGLO (ratio) | 1.39 (1.19-1.58) | 1.37 (1.12-1.56) | 0.175 |
| ALP (U/L) | 83 (65.8-103.5) | 78.55 (63.55-101.75) | 0.329 |
| UA (umol/L) | 313.4 (257.25-383.65) | 321.05 (245.13-404.5) | 0.672 |
| LDL (mmol/L) | 2.65 (2.17-3.37) | 2.81 (2.29-3.45) | 0.156 |
| TC (mmol/L) | 4.59 (3.93-5.58) | 4.86 (4.06-5.44) | 0.577 |
| TBIL (umol/L) | 10.8 (8.3-13.8) | 10.55 (8.013-13.76) | 0.575 |
| apoAI (g/L) | 1.167 (0.94-1.39) | 1.155 (0.91-1.38) | 0.825 |
| TG (mmol/L) | 1.1 (0.84-1.68) | 1.27 (0.91-1.96) | 0.059 |
| **Medical history** |  |  |  |
| Diabetes | 73 (21.0%) | 31 (20.7%) | >0.99 |
| Hypertension | 163 (47.0%) | 73 (48.7%) | 0.803 |
| **Complications*** |  |  |  |
| Parkinson's disease | 0 (0%) | 1 (0.37%) | >0.99 |
| Alzheimer's disease | 0 (0%) | 1 (0.37%) | >0.99 |
| NSAIDs-induced gastrointestinal bleeding | 0 (0%) | 0 (0%) | >0.99 |

Abbreviations: Lymph: lymphocyte; Neut: neutrophil; Ca: calcium; ALT: alanine transaminase; Na: serum sodium; ApoB: Apolipoprotein B; Crea: Creatinine; HDL: High-density lipoprotein cholesterol; AST: aspartate aminotransferase; ALBGLO: albumin to globulin ratio; ALP: alkaline phosphatase; UA: uric acid; LDL: low-density lipoproteins; TC: total cholesterol; TBIL: total bilirubin; apoAI: Apolipoprotein A-I; TG: triglyceride; NSAIDS: nonsteroidal anti-inflammatory drugs

#age is the only variable that follows a normal distribution; thus, it is presented as mean (SD)

*Due to a low variance, these variables were excluded from modeling, but were not excluded from descriptive analysis.

**Table S3**. Performance of each algorithm on training and validation data.

| **Algorithm** | **AUC (95% C.I.)** | |
| --- | --- | --- |
|  | **Training**  **(N = 347)** | **Validation**  **(N = 150)** |
| MLR | 0.724 [0.671, 0.778] | 0.631 [0.541, 0.72] |
| Lasso | 0.653 [0.595, 0.711] | 0.623 [0.535, 0.716] |
| Random Forest | 1 [1, 1] | 0.664 [0.579, 0.754] |
| XGBoost | 0.795 [0.748, 0.841] | 0.667 [0.583, 0.761] |
| SuperLearner | 0.942 [0.920, 0.965] | 0.677 [0.591, 0.762] |

Abbreviations: AUC: area under curve; MLR: multiple logistic regression; Lasso: Least Absolute Shrinkage and Selection Operator; XGBoost: Extreme Gradient Boosting

**Table S4**. Summary of AUC values from random partitioning and bootstrapping.

|  | **Min** | **25%** | **Median** | **Mean** | **75%** | **Max** |
| --- | --- | --- | --- | --- | --- | --- |
| Partitioning | 0.5571 | 0.6347 | 0.6571 | 0.6594 | 0.6882 | 0.7429 |
| Bootstrap | 0.5994 | 0.6397 | 0.6599 | 0.6591 | 0.6801 | 0.7319 |
